# Pulmonary Function and Incident Bronchitis and Asthma in Children: A Community-Based Prospective Cohort Study

**DOI:** 10.1371/journal.pone.0032477

**Published:** 2012-03-23

**Authors:** Yungling Leo Lee, Bing-Fang Hwang, Yu-An Chen, Jer-Min Chen, Yi-Fan Wu

**Affiliations:** 1 Institute of Epidemiology and Preventive Medicine, College of Public Health, National Taiwan University, Taipei, Taiwan; 2 Department of Public Health, College of Public Health, National Taiwan University, Taipei, Taiwan; 3 Department of Occupational Safety and Health, College of Public Health, China Medical University, Taichung, Taiwan; 4 Department of Family Medicine, Taipei City Hospital, Renai Branch, Taipei, Taiwan; Abramson Research Center, United States of America

## Abstract

**Background:**

Previous studies revealed that reduction of airway caliber in infancy might increase the risks for wheezing and asthma. However, the evidence for the predictive effects of pulmonary function on respiratory health in children was still inconsistent.

**Methods:**

We conducted a population-based prospective cohort study among children in 14 Taiwanese communities. There were 3,160 children completed pulmonary function tests in 2007 and follow-up questionnaire in 2009. Poisson regression models were performed to estimate the effect of pulmonary function on the development of bronchitis and asthma.

**Results:**

After adjustment for potential confounders, pulmonary function indices consistently showed protective effects on respiratory diseases in children. The incidence rate ratios of bronchitis and asthma were 0.86 (95% CI 0.79–0.95) and 0.91 (95% CI 0.82–0.99) for forced expiratory volume in 1 second (FEV_1_). Similar adverse effects of maximal mid-expiratory flow (MMEF) were also observed on bronchitis (RR = 0.73, 95% CI 0.67–0.81) and asthma (RR = 0.85, 95% CI 0.77–0.93). We found significant decreasing trends in categorized FEV_1_ (*p* for trend = 0.02) and categories of MMEF (*p* for trend = 0.01) for incident bronchitis. Significant modification effects of traffic-related air pollution were noted for FEV_1_ and MMEF on bronchitis and also for MMEF on asthma.

**Conclusions:**

Children with high pulmonary function would have lower risks on the development of bronchitis and asthma. The protective effect of high pulmonary function would be modified by traffic-related air pollution exposure.

## Introduction

Previous researches showed that bronchitis and asthma were substantial respiratory health issues in children, which may cause major childhood morbidity and high socioeconomic costs [1], [2]. Some studies have suggested a complex etiological pathway for certain respiratory diseases but until now, definite pathogenesis was still unclear [3]. There is growing evidence that air pollution is an important environmental factor that may be associated with childhood asthma [4], [5]. Children with higher prevalence of bronchitis resided in areas with higher ambient air pollutants, such as nitrogen dioxide (NO_2_), has also been reported [6], [7]. In the previous finding from Taiwan Children Health Study (TCHS), we found that risks of the microsomal epoxide hydroxylase (*EPHX1*) gene for childhood respiratory health could be modified by ambient NO_2_ levels [8], which suggested that air pollution may overwhelm the genetic effects and increase the risk of respiratory diseases in a subset of children.

Pulmonary function is a sensitive marker of respiratory health, which can detect and grade airway obstruction [9]. Bronchial hyper-reactivity was an obvious characteristic of asthma and could lead to the bronchial symptoms in both children and adults [10], [11]. Previous studies showed that high pulmonary function indices such as the ratio of maximal mid-expiratory flow divided by forced vital capacity (MMEF/FVC) and MMEF were inversely associated with bronchial hyper-reactivity [12], [13]. Although the association between pulmonary function during the first few weeks of life and onset of asthma in later life had been reported before [14]–[16], evidence for the effect of pulmonary function on incident respiratory diseases in school-aged children was inconclusive.

TCHS was a community-based longitudinal study that provided information about genetics, respiratory health and environmental factors, such as ambient air pollution. We had the opportunity to assess the hypothesis that high pulmonary function indices are associated with reduced risk of incident respiratory diseases. The modification effect for traffic-related air pollution could also be examined in the present study.

## Materials and Methods

### Ethics approval

The study protocol was approved by the institutional review board of our university hospital and complied with the principles outlined in the Helsinki Declaration [17].

**Figure 1:**
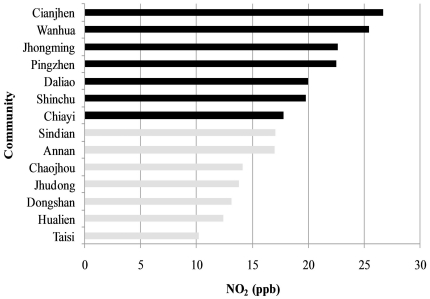
Annual average NO_2_ levels (2007 –**2009) in 14 Taiwan Children Health Study communities.** Dark bars: higher NO_2_; mean, 22.1 ppb. Light bars: lower NO_2_; mean, 14.0 ppb.

### Study design

TCHS is a prospective cohort study examining the determinants of children's respiratory health, which focused on outdoor air pollutants as primary interest. The study design and methods for TCHS have been described in detail previously [7], [18]. A total of 4,134 seventh-grade children were recruited from public schools from 14 communities. At cohort entry in 2007, all participants were collected with baseline respiratory conditions and arranged to complete pulmonary function tests. We followed these children in 2009 with the identical questions concerning respiratory diseases and pulmonary function tests. The mean follow-up period for the current study was two years.

Questions regarding respiratory symptoms and diseases were modified after those used in the Children's Health Study in southern California [19]. The outcomes of interest were physician-diagnosed bronchitis and asthma. Children who were disease-free at baseline and reported a yes answer to the question “Has a physician ever diagnosed your child as having bronchitis?” on follow-up questionnaire were classified as having incident bronchitis. The incident asthma was defined by the question “Has a physician ever diagnosed your child as having asthma?” on baseline and follow-up questionnaire. Those with wheezing history but no diagnosis were considered to be at risk for a new diagnosis of bronchitis or asthma and were included in the study.

### Pulmonary function measurement

Children's pulmonary function tests (PFT) were performed during the morning hours in indoor buildings to avoid daily and annual peak air pollution levels. For the baseline survey in 2007, children returned parental questionnaire were included. After excluding subjects with incomplete questionnaire, recent symptomatic upper respiratory infections, or other acute pulmonary function or cardiac diseases, there were 3,261 children eligible for pulmonary function tests at the baseline survey.

Each subject was requested to perform three satisfactory blows, defined as both of the two largest FVC and forced expiratory volume in 1 second (FEV_1_) agreeing within 150 ml, extrapolation volume less than 150 ml or 5% of FVC, and forced expiratory time exceeding 6 seconds. These criteria were based on American Thoracic Society and European Respiratory Society recommendations, updated in 2005, modified for children [20]. No more than 5 blows were attempted per time, no more than two times were asked per child. Resting for more than 10 minutes was required for every subject to prevent exercise bias. To predict subjects' pulmonary function, height and weight were measured at the time of testing. Two fully-trained technicians performed PFT, using two spirometers (Chestgraph HI-101, CHEST M.I., INC). Spirometers' calibrations were checked before, during, and after every morning's testing using 1L flow-volume syringes.

Three pulmonary function indices were analyzed: FVC, FEV_1_ and MMEF. We obtained sex-specific percentage predicted pulmonary function indices by using linear regression models. The selection of the best prediction models from age, height, nature log values for height, the square of height, weight, nature log values for weight, the square of weight, body-mass index, nature log values for body-mass index and the square of body-mass index was based on the attained adjusted R^2^ using sex-specific equations (Table S1). We calculated each percentage predicted pulmonary function index from dividing the observed values by the predicted values and was expressed in percentage. Then, each percentage predicted pulmonary function index was categorized into three groups based on the cut-off points 90% and 110% that provided an adequate distribution for analyses.

**Table 1:** Demographic characteristics of the study participants.

Selected characteristics at study entry^a^	Bronchitis-free cohort		Asthma-free cohort	
	(N = 2,893)		(N = 2,818)	
	n	%	n	%
Gender				
Boys	1466	51.5	1452	51.3
Girls	1380	48.5	1352	48.7
Age, yr				
12	2254	79.5	2200	79.5
13	576	20.3	561	20.3
14	5	0.2	5	0.2
Parental income^b^				
<$>\scale 85%\raster="rg1"<$>400,000	943	35.9	930	36.3
410,000∼800,000	1059	40.4	1031	40.3
<$>\scale 75%\raster="rg2"<$>810,000	622	23.7	598	23.4
Parental education, yr				
<$>\scale 85%\raster="rg1"<$>12	1860	65.4	1813	65.3
13∼15	554	19.5	547	19.7
<$>\scale 75%\raster="rg2"<$>16	432	15.2	417	15.0
Family history of asthma				
Yes	69	2.4	55	2.0
Family history of atopy^c^				
Yes	663	23.3	655	23.6
*In utero* exposure to maternal smoking				
Yes	110	3.9	103	3.7
Active smoking				
Yes	149	5.2	145	5.2
Current SHS				
Yes	1222	42.9	1209	43.5
Carpet use				
Yes	254	9.0	252	9.1
Dogs in home				
Yes	807	28.4	784	28.2
Cats in home				
Yes	149	5.2	146	5.3
Cockroaches				
Yes	2413	85.5	2367	85.9
Visible mould				
Yes	963	34.1	957	34.7
Mildewy odor				
Yes	416	14.7	413	14.9
Water stamp on the wall				
Yes	718	25.4	711	25.7

a Number of subjects do not add up to total N because of missing data.

b New Taiwan dollars per year ($1 US = $ 33 New Taiwan).

c Atopy is defined as allergic rhinitis or atopic eczema.

### Air pollution monitoring data

Complete monitoring data for the criteria air pollutants are available from Taiwan Environmental Protection Agency (EPA) monitoring stations beginning in 1995. The average hourly levels of NO_2_ were measured in 14 monitoring stations. We computed the annual average of ambient NO_2_ levels between 2007 and 2009. The 14 communities were grouped by median NO_2_ level into seven higher and seven lower ones (median level, 17.5 ppb). The mean annual ambient NO_2_ level was 22.1 ppb in higher NO_2_ communities and 14.0 ppb in lower NO_2_ communities (Figure 1).

### Statistical analysis

The association between pulmonary function indices and the incidence of bronchitis and asthma were examined by fitting Poisson regression models (PROC GENMOD) with a logarithmic link function. To account for over-dispersion of incidence rates in Poisson models, we performed all analyses by using deviance adjusted variance estimates [21]. On the basis of study design and *a priori* consideration of potential confounders, we included *in utero* exposure to maternal smoking, family history of asthma, family history of atopy, and community in all models. If estimates of pulmonary function indices changed by more than 10% when a covariate was added in base models, the covariate would be included in final models [22]. Subjects with missing covariate information were included in the model using missing indicators [23]. The incidence rate ratios were scaled by inter-quartile range (IQR) increase of the corresponding values in certain pulmonary function indices. We also conducted sensitivity analyses by restricting the cohort to children without a history of bronchitis, asthma or wheezing at study entry.

The influences of community-level air pollutants were estimated by fitting two-stage hierarchical models. The models assumed two sources of variation: the variation among subjects in the first stage, part of which could be explained by the individual confounders, and the variation of air pollution between communities in the second stage, part of which could be explained by variables measured at the community level. To investigate the effects of traffic-related air pollution on the relationship between pulmonary function indices and respiratory diseases, we conducted stratified analysis comparing the children in communities with lower and higher levels of NO_2_. We used likelihood ratio tests to examine the interactive effects between pulmonary function indices and ambient NO_2_ levels on respiratory diseases.

We also presented graphically the potential modification effects of traffic-related air pollution by plotting ambient NO_2_ levels on X-axis and community-specific rate ratios on Y-axis. The regression curves were drawn through the predicted values derived from exponential regression models. All analyses were performed by SAS software version 9.1 (SAS Institute, Gary, NC, USA) and assumed a 0.05 significant level based on a two-sided estimate.

**Table 2:** Association between incident bronchitis and asthma and pulmonary function indices at study entry.

	Bronchitis		Asthma	
Pulmonary function index^a^	RR^b^	95% CI	RR^b^	95% CI
FVC (% predicted)	1.08	0.99 – 1.18	0.90	0.82 – 0.99
FEV_1_ (% predicted)	0.86	0.79 – 0.95	0.91	0.82 – 0.99
MMEF (% predicted)	0.73	0.67 – 0.81	0.85	0.77 – 0.93

a FVC, forced vital capacity; FEV_1_, forced expiratory volume in 1s; MMEF, forced expiratory flow over the mid-range of expiration.

b Relative risks (RR) and 95% confidence intervals (CI) of outcomes were scaled across the inter-quartile range elevation by each pulmonary function index.

All models were adjusted for community, *in utero* exposure to maternal smoking, family history of asthma, family history of atopy, active smoking and current SHS.

## Results

### Participant characteristics

In 2009, a total of 3,160 children completed the follow-up questionnaire and pulmonary function tests, with follow-up rate of 96.9%. There were 2,893 bronchitis-free children and 2,818 asthma-free children at cohort entry. The incidence rates were 8.1/1000 person-years for bronchitis and 7.2/1000 person-years for asthma. The characteristics of study participants were shown in Table 1. The proportion of gender was almost equal (51% male and 49% female). Approximately 24% of the children had family history of atopy which included any history of hay fever, allergies to food or medicine, inhaled dusts, pollen, molds, animal fur or dander, or skin allergies. The prevalence of current second-hand smoke (SHS) and active smoke exposure were around 43% and more than 5% among children. The distribution of all covariates was consistent in the two disease-free cohorts.

### Pulmonary function and respiratory diseases

The relationships between pulmonary function indices and respiratory diseases were presented in Table 2. We found the consistently protective effects for all pulmonary function indices. Scaled across inter-quartile range elevation, the incidence rate ratio of bronchitis was 0.86 (95% CI 0.79−0.95) for FEV_1_ and 0.73 (95% CI 0.67−0.81) for MMEF. Similar adverse effects were observed for asthma with incidence rate ratio 0.90 (95% CI 0.82−0.99) for FVC, 0.91 (95% CI 0.82−0.99) for FEV_1_, and 0.85 (95% CI 0.77−0.93) for MMEF. Restricting the analyses to children without a history of bronchitis, asthma or wheezing did not substantially alter the above findings (Table S2).

**Table 3:** Association between incident bronchitis and asthma and pulmonary function indices categories at study entry.

	Bronchitis				Asthma			
Pulmonary function index*	RR	95% CI			RR	95% CI		
FVC (% predicted)								
<$>\scale 85%\raster="rg1"<$>90	1.00				1.00			
90–110	1.55	1.16 – 2.06			0.93	0.71 – 1.22		
<$>\scale 75%\raster="rg2"<$>110	0.78	0.52 – 1.16			0.72	0.50 – 1.03		
*p* for trend	0.38				0.08			
FEV_1_ (% predicted)								
<$>\scale 85%\raster="rg1"<$>90	1.00				1.00			
90–110	0.80	0.63 – 1.02			0.57	0.45 – 0.76		
<$>\scale 75%\raster="rg2"<$>110	0.68	0.49 – 0.95			0.70	0.51 – 0.95		
*p* for trend	0.02				0.01			
MMEF (% predicted)								
<$>\scale 85%\raster="rg1"<$>90	1.00				1.00			
90–110	0.60	0.48 – 0.75			0.78	0.61 – 1.00		
<$>\scale 75%\raster="rg2"<$>110	0.44	0.33 – 0.57			0.92	0.71 – 1.18		
*p* for trend	0.01				0.47			

*FVC, forced vital capacity;

FEV_1_, forced expiratory volume in 1s;

MMEF, forced expiratory flow over the mid-range of expiration.

All models were adjusted for community, *in utero* exposure to maternal smoking, family history of asthma, family history of atopy, active smoking and current SHS.

Table 3 presents the associations between pulmonary function indices categories and incident bronchitis and asthma. The incidence rate ratios for bronchitis and asthma were decreased in the upper categories. In bronchitis-free cohort, significant decreasing trends were found on categories of FEV_1_ (*p* for trend = 0.02) and on categories of MMEF (*p* for trend = 0.01).

**Table 4:** Relative risks of incident bronchitis and asthma by pulmonary function indices, stratified by community-specific annual average NO_2_ levels (2007–2009).

	Bronchitis					Asthma				
	Low NO_2_ b		High NO_2_ b		interaction ***p*** value	Low NO_2_ b		High NO_2_ b		interaction ***p*** value
Pulmonary function index^a^	RR^c^	95% CI	RR^c^	95% CI		RR^c^	95% CI	RR^c^	95% CI	
FVC (% predicted)	0.83	0.72 – 0.95	1.25	1.10 – 1.42	0.12	0.82	0.72 – 0.93	1.00	0.88 – 1.14	0.40
FEV_1_ (% predicted)	0.56	0.49 – 0.65	1.10	0.96 – 1.24	0.005	0.85	0.75 – 0.97	0.94	0.82 – 1.08	0.47
MMEF (% predicted)	0.43	0.37 – 0.50	0.96	0.85 – 1.10	<0.001	0.73	0.64 – 0.83	0.97	0.85 – 1.11	0.04

a FVC, forced vital capacity; FEV_1_, forced expiratory volume in 1s; MMEF, forced expiratory flow over the mid-range of expiration.

b Two NO_2_ strata were defined as less than and greater than the median level (17.5 ppb).

c Relative risks (RR) and 95% confidence intervals (CI) of outcomes were scaled across the inter-quartile range elevation by each pulmonary function index.

All models were adjusted for community, *in utero* exposure to maternal smoking, family history of asthma, family history of atopy, active smoking and current SHS.

### Interrelationship between traffic-related air pollution, pulmonary function and respiratory diseases

**Figure 2:**
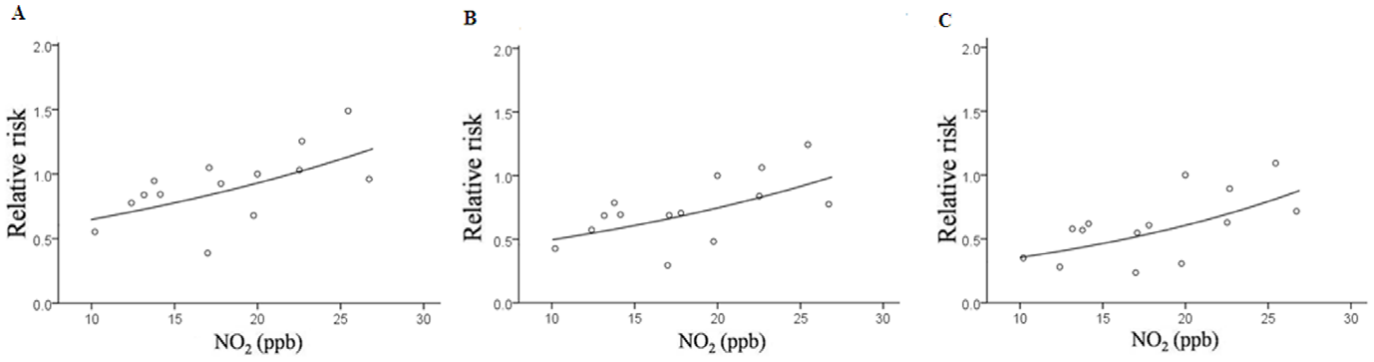
Community-specific relative risks (RR) of incident bronchitis across inter-quartile range elevation for pulmonary function indices by ambient NO_2_ levels. Each circle represents one community. (A) FVC, forced vital capacity; (B) FEV_1_, forced expiratory volume in 1s; (C) MMEF, forced expiratory flow over the mid-range of expiration.

We found loss of protective effects by each pulmonary function index against bronchitis, chronic cough and asthma in the higher NO_2_ communities (Table 4). Scaled across the inter-quartile range elevation of FEV_1_, the incidence rate ratio of bronchitis was 0.56 (95% CI 0.49–0.65) in the lower NO_2_ communities, whereas the effect was 1.10 (95% CI 0.96–1.24) in the higher NO_2_ communities (*p* for interaction = 0.005). For each inter-quartile range changes in MMEF, the effects on incident bronchitis were 0.43 (95% CI 0.37–0.50) in the lower NO_2_ communities and 0.96 (95% CI 0.85–1.10) in the higher NO_2_ communities (*p* for interaction <0.001). Similarly, the incidence rate ratio of asthma was significantly lower in the lower NO_2_ communities (RR = 0.73, 95% CI 0.64–0.83), compared with the effect in the higher NO_2_ communities (RR = 0.97, 95% CI 0.85–1.11) (*p* for interaction = 0.04) (Table 4). In addition, we found no statistical significant differences on the effects of pulmonary function indices for incident bronchitis in relation to exposure to the other air pollutants in TCHS (PM_2.5_, PM_10_ and 8-hour O_3_). We calculated the communities-specific rate ratios of each pulmonary function index on incident bronchitis. The loss of protective effects from high pulmonary function indices would be interpreted through Figure 2, with significant interactions between pulmonary function indices and ambient NO_2_ levels. We found the protective effects would disappear in communities over ambient NO_2_ level of 25 ppb.

## Discussion

We examined the relationship between pulmonary function and respiratory diseases among children under different traffic-related air pollutant levels. Pulmonary function indices, such as FEV_1_ and MMEF, were found to predict the new-onset bronchitis and asthma. Exposure to ambient NO_2_ would reduce the protective effect of high pulmonary function on the occurrence of bronchitis. To our best knowledge, this is the first report to describe the association between pulmonary function indices and incident bronchitis.

Our work is a prospective cohort study with a very high follow-up rate (96.9%). The participants were representative of the native population of Taiwan. Some individual characteristics, such as *in utero* exposure to maternal smoking [24], [25], family history of asthma or atopy [26], [27], and residential community may influence the occurrence of asthma in childhood. Active smoking and current SHS were also found to interfere in the association with respiratory health [28]–[32]. We adjusted the above covariates in Poisson regression models to minimize the confounding effects.

Some studies assessed the role of pulmonary function indices in respiratory health. The airway caliber in infancy has been reported as a decreasing risk for wheezing [16]. The association between the reduced airway function in the first few weeks of life and asthma in later life had also been described [14], 15. In previous epidemiological studies, high flow rates were noted to be significantly correlated with a low prevalence of airway hyper-reactivity [13], [33], and age-related decline on prevalence of airway hyper-reactivity was larger among those with higher FEV1% [34]. Furthermore, Islam *et al*. reported that high pulmonary function provided protective effects in asthma development during childhood [35]. The relationship of pulmonary function indices on development of bronchitis in children has not been previously investigated.

Pulmonary function indices have been clinically proved as important phenotypes of respiratory health. Bronchial hyper-reactivity could lead to the respiratory symptoms in childhood [10], [11]. In a previous cross-sectional study from the United States, methacholine airway responsiveness was noted to be inversely associated with high pulmonary function indices such as MMEF/FVC [12]. Another hospital-based study from Italy also revealed that MMEF was related to bronchial hyper-reactivity consistently among patients with asthma and rhinitis [13]. All the above evidences were consistent with our finding that pulmonary function indices could well predict the occurrence of respiratory diseases in childhood.

The respiratory system is particularly sensitive to air pollutants. Traffic-related air pollution has been reported as a key factor for the incidence of respiratory diseases [36]. NO_2_, the representative traffic-related air pollutant, is through interaction with impairment of respiratory response or the immune system to infection, and damages the epithelial cells by oxidative injury [7], [37], [38]. In Taiwan, we have previously reported that an increase of 8.79 ppb of ambient NO_2_ exposure would result in 80% increase in the prevalence of bronchitic symptoms [7]. We have interpreted our data as a protective effect of high pulmonary function that was attenuated by the effects of traffic-related air pollution (Table 4). This finding was also consistent with previous analyses in our cohort that the risk of respiratory morbidity associated with the *EPHX1* 139Arg allele could be modified by ambient NO_2_ levels [8]. The pathogenesis of bronchitis and asthma included airway remodeling characterized by extraordinary mucous membrane secretion and mild bronchial wall thickening. Long-term exposure to higher level of air pollution has been noted to be associated with small airway remodeling, followed by chronic bronchial obstruction [39]. It is likely that evolutionary selection has resulted in pulmonary characteristics that lead to better respiratory health. Higher pulmonary function may be a marker for lower susceptibility to respiratory diseases.

Another concern for present study was the insufficient power to detect relatively small modification effects of air pollution on incident asthma. The primary pathophysiological characteristics of bronchitis may include chronic pulmonary inflammation and cellular oxidative stress [36], which might result from chemical irritants such as NO_2_. The relatively higher predictive power for bronchitis rather than asthma may be due to the essentially different pathophysiology between these two kinds of diseases.

Our study has some limitations. The assessment of respiratory diseases was based on questionnaire reports. Nevertheless, questionnaire was widely used to define respiratory outcomes in epidemiologic studies in children. Bronchitis and asthma were through physician-diagnoses, which would reduce the possibility of differential symptom identifications and recall bias. Another limitation was the measurements of pulmonary function depending on many known or unknown factors, such as the quality of the spirometry equipment, technician's skill, and children's cooperation [9]. In order to avoid the possible errors of PFT measurements, the children's PFT were performed by the same well-trained technicians and spirometers in indoor building within the same season. We minimized these potential information biases in our study design. It is possible that the cases of new-onset bronchitis or asthma in our study were undiagnosed cases and had low pulmonary function at study entry. This seems unlikely because effect estimates did not changed substantially when we restricted the analyses to children without a history of bronchitis, asthma or wheezing. Because incident cases were defined without knowledge of pulmonary function measurement, differential misclassification is probably not a major source of bias that accounts for our results.

In conclusion, our study provides new evidence for the protective effects of high pulmonary function indices on incidence of bronchitis and asthma. What is important to investigate is that traffic-related air pollution is a substantial modifiable risk for childhood respiratory diseases. Children living in communities with higher traffic-related air pollution would suffer from increased risks for new-onset bronchitis and asthma than those in lower polluted communities. Additional research is necessary to clarify the mechanism by NO_2_ modifies the protective effect of high pulmonary function on the development of bronchitis and asthma.

## Supporting Information

Table S1 Predictive equations proposed in this study using age, height, weight, and body mass index variables.
(DOC)

Table S2 Association between incident bronchitis and asthma and pulmonary function indices, after excluding those with bronchitis, asthma or wheezing at study entry.
(DOC)
