# Supplementary material for: Pulmonary Function and Incident Bronchitis and Asthma in Children: A Community-Based Prospective Cohort Study
Source: PLoS One. 2012 Mar 23;7(3):e32477. doi: 10.1371/journal.pone.0032477 (PMC3311633; doi:10.1371/journal.pone.0032477)
Supplement: Table S1 — Predictive equations proposed in this study using age, height, weight, and body mass index variables. (DOC) [file pone.0032477.s001.doc]

| **Table S1: Predictive equations proposed in this study using age, height, weight, and body mass index variables** | | | |
| --- | --- | --- | --- |
| **Pulmonary function index** |  | **Equation** | |
| Boys | |  |  |
| FVC (mL) | | = 279938 + (3.3 × A) + (1250.8 × H) + (-84708 × lnH) + (-1.8 × H^2^) + (30.1 × W) + (-8235.2 × lnW) + (-325.9 × B) + (12215 × lnB) + (1.9 × B^2^) |  |
| FEV1 (mL) | | = 301313 + (16.7 × A) + (1271.3 × H) + (-89975 × lnH) + (-1.9 × H^2^) + (16.5 × W) + (-6278.2 × lnW) + (-373.4 × B) + (10782 × lnB) + (2.9 × B^2^) |  |
| MMEF (mL/s) | | = 477587 + (51.5 × A) + (1749.3 × H) + (-138131 × lnH) + (-2.6 × H^2^) + (-655.7 × B) + (7901.6 × lnB) + (6.4 × B^2^) |  |
|  | |  |  |
| Girls | |  |  |
| FVC (mL) | | = -9433.6 + (-0.2 × A) + (78.7 × H) + (-4229.4 × lnW) + (0.1 × W^2^) + (-85.6 × B) + (5882.5 × lnB) |  |
| FEV1 (mL) | | = -57009 + (12.5 × A) + (12344 × lnH) + (27.2 × W) + (-5305.4 × lnW) + (-80 × B) + (5947 × lnB) |  |
| MMEF (mL/s) | | = 2217 + (51 × A) + (40.4 × W) + (-86.6 × B) |  |
| FVC, forced vital capacity; FEV_1_, forced expiratory volume in 1s; MMEF, forced expiratory flow over the mid-range of expiration.  W = weight (kg), H = height (cm), A = age (years), B= body mass index (kg/m^2^). | | | |
| The selection of the best prediction model was based on the attained adjusted R^2^. | | | |
