# Supplementary material for: Pulmonary Function and Incident Bronchitis and Asthma in Children: A Community-Based Prospective Cohort Study
Source: PLoS One. 2012 Mar 23;7(3):e32477. doi: 10.1371/journal.pone.0032477 (PMC3311633; doi:10.1371/journal.pone.0032477)
Supplement: Table S2 — Association between incident bronchitis and asthma and pulmonary function indices, after excluding those with bronchitis, asthma or wheezing at study entry. (DOC) [file pone.0032477.s002.doc]

| **Table S2: Association between incident bronchitis and asthma and pulmonary function indices, after excluding those with bronchitis, asthma or wheezing at study entry** | | | | | | | | | | |
| --- | --- | --- | --- | --- | --- | --- | --- | --- | --- | --- |
|  | **Bronchitis** | | | |  | **Asthma** | | | |  |
| **Pulmonary function index^a^** | **RR^b^** | **95% CI^b^** | | |  | **RR^b^** | **95% CI^b^** | | |  |
| FVC (% predicted) | 1.06 | 0.96 | - | 1.17 |  | 0.93 | 0.84 | - | 1.03 |  |
| FEV_1_ (% predicted) | 0.90 | 0.81 | - | 0.99 |  | 0.96 | 0.86 | - | 1.06 |  |
| MMEF (% predicted) | 0.91 | 0.81 | - | 0.99 |  | 0.89 | 0.81 | - | 0.99 |  |
| ^a^ FVC, forced vital capacity; FEV_1_, forced expiratory volume in 1s; MMEF, forced expiratory flow over the mid-range of expiration. | | | | | | | | | | |
| ^b^ Relative risks (RR) and 95% confidence intervals (CI) of outcomes were scaled across the inter-quartile range elevation by each pulmonary function index. | | | | | | | | | | |
| All models were adjusted for community, *in utero* exposure to maternal smoking, family history of asthma, family history of atopy, active smoking and current SHS. | | | | | | | | | | |
